# Pancreatoduodenal Tuberculosis: A Rare Site of a Common Disease

**DOI:** 10.7759/cureus.23275

**Published:** 2022-03-17

**Authors:** Arvind Singh, Archana Khanduri, Nalini Bansal, Pradip Pokharia, Rahul Gupta

**Affiliations:** 1 Gastroenterology, Synergy Institute of Medical Sciences, Dehradun, IND; 2 Gastrointestinal Surgery, Synergy Institute of Medical Sciences, Dehradun, IND; 3 Histopathology, SRL Diagnostics, Fortis Escorts Heart Institute, New Delhi, IND; 4 Radiology, Synergy Institute of Medical Sciences, Dehradun, IND

**Keywords:** non-caseating granuloma, endoscopic ultrasound (eus), endoscopy, abscess, duodenum, pancreas, tuberculosis

## Abstract

The most common site of abdominal tuberculosis (TB) is the ileocecal region. The duodenum and pancreas are rare sites of abdominal TB. It is usually observed in immunocompromised patients with miliary or disseminated TB. Pancreatoduodenal TB is often misdiagnosed as malignancy due to a lack of specific symptoms. Here, we present a case of a 55-year-old immunocompetent man having abdominal pain, bilious vomiting, weight loss, and high-grade fever for three months. Contrast-enhanced computed tomography of the abdomen showed a localized abscess with air foci in the pancreatic head and duodenopancreatic groove. Additionally, there was retroperitoneal lymphadenopathy and mild ascites. Upper gastrointestinal endoscopy revealed an ulceroproliferative lesion in the second part of the duodenum suspicious of a malignant tumor. However, the endoscopic biopsy showed epitheloid-like histiocytes, Langerhans type of giant cells, and granuloma formation suggestive of TB. The patient responded to antitubercular treatment and became symptom-free.

## Introduction

Tuberculosis (TB) is a major public health problem worldwide, especially in developing countries. Although lungs are the primary site of involvement in most cases, extrapulmonary TB is found in 12-15% of immunocompetent patients [1]. Abdominal TB includes the involvement of the gastrointestinal tract, peritoneum, lymph nodes, and solid organs of the abdomen. In the gastrointestinal tract, the ileocecal region is the most prominent site for TB. Non-specific features of abdominal TB results in delayed diagnosis. Risk factors for abdominal TB include malnutrition, liver cirrhosis, diabetes mellitus, immunocompromised status such as human immunodeficiency virus (HIV) infection, and use of immunosuppression. The duodenum and pancreas are rare sites of gastrointestinal TB [2-6]. The reported incidence of duodenopancreatic TB varies between 0% to 8% [7-9]. However, in recent times, the incidence of pancreatic TB is increasing. The diagnosis of pancreatoduodenal TB is often challenging as the clinical features are similar to that of common gastrointestinal diseases such as peptic ulcer disease and malignancy. We report a case of a 55-year-old male with endoscopic and radiological findings suspicious of malignancy but found to have pancreatoduodenal TB on histopathological examination of the endoscopic biopsy.

## Case presentation

A 55-year-old man having type II diabetes mellitus and hypothyroidism with no past or family history of TB presented with complaints of high-grade fever and weight loss for three months. The patient experienced intermittent high-grade fever, which subsided with antipyretics. Over the last three months, he had 20 kg of unintentional weight loss. Subsequently, he developed abdominal pain and bilious vomiting for 10 days. Clinical examination was unremarkable. Blood investigations revealed mild anemia, hyponatremia, hypocalcemia (Table 1). Liver function tests and tumor markers were within normal limits (Table 1). 

**Table 1:** Laboratory parameters. CA 19.9: carbohydrate antigen 19.9; CEA: carcinoembryonic antigen

Parameters	Patient’s value	Reference range
Hemoglobin (gm/dl)	10	11-15
Total leucocyte count (cells/cu.mm.)	5590	4000-11000
Platelet count (lakh/cu.mm)	3.76	1.5-4.5
Total bilirubin (mg/dl)	0.9	0.1-1.2
Direct bilirubin (mg/dl)	0.4	0-0.2
Aspartate aminotransferase (IU/L)	37	0-40
Alanine aminotransferase (IU/L)	41	10-50
Alkaline phosphatase (IU/L)	156	40-130
Serum albumin (gm/dl)	3.4	3.97-4.94
Serum creatinine (mg/dl)	0.8	0.3-1.3
Serum sodium (mmol/L)	124	136-145
Serum potassium (mmol/L)	4.6	3.5-5.5
Serum calcium (mg/dl)	8.6	8.7-10.2
CA 19.9 (U/ml)	20.8	0-37
CEA (ng/ml)	1.2	0-2.5

**Figure 1:**
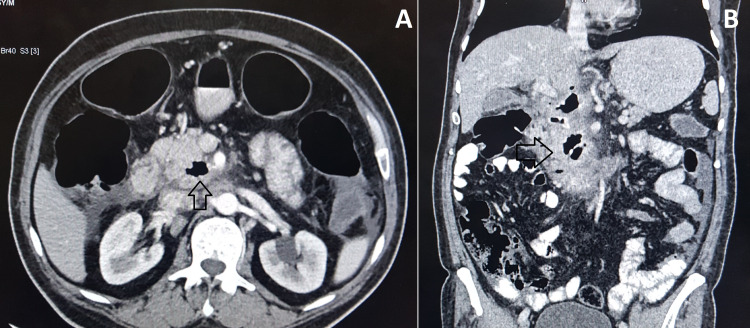
Contrast-enhanced computed tomography of the abdomen showing the hypodense lesion containing air foci (arrows) in the pancreatic head region with surrounding fat stranding in the axial (A) and coronal (B) sections.

Contrast-enhanced computed tomography (CECT) of the abdomen showed a hypodense lesion of 3.6 x 4 x 4.4 cm with air foci in the pancreatic head and duodenopancreatic groove suggestive of localized abscess with duodenal thickening, retroperitoneal lymphadenopathy, and mild ascites (Figure 1). CECT chest revealed mediastinal lymphadenopathy. 

**Figure 2:**
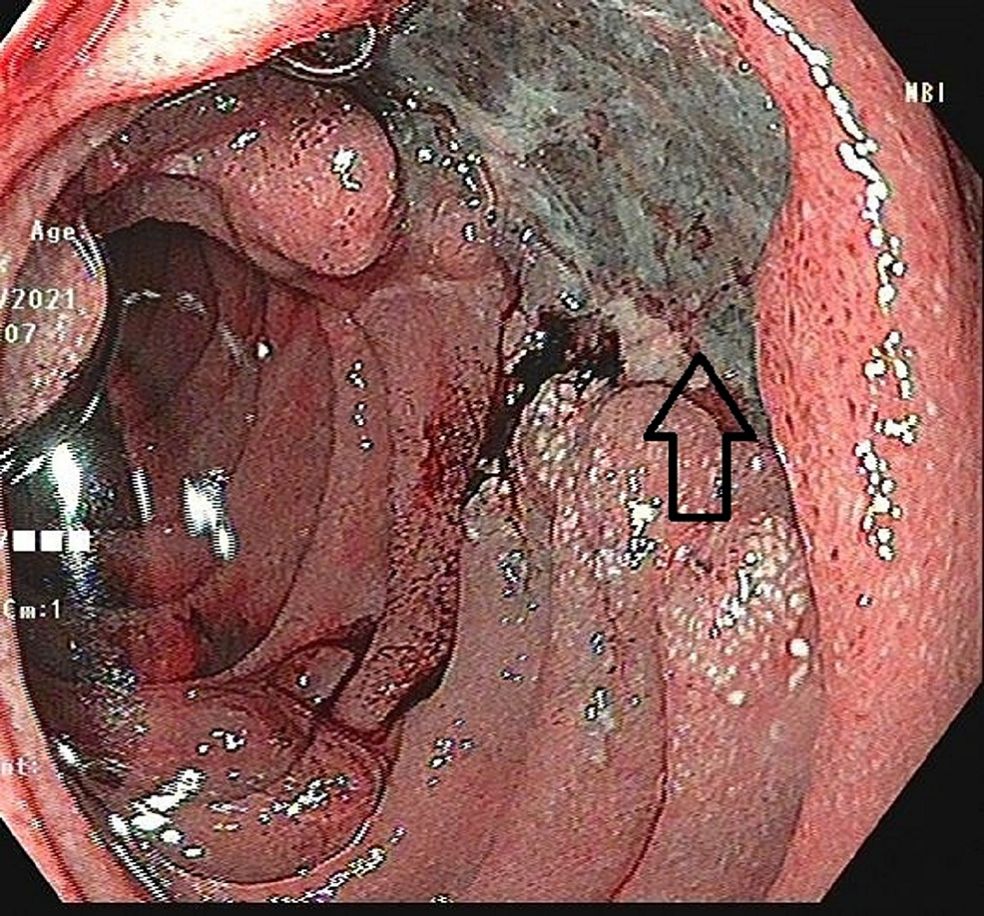
Upper gastrointestinal endoscopy showing an ulcer with nodular margins in the medial wall of the second part of the duodenum near the papilla (arrow).

Upper gastrointestinal endoscopy (UGIE) found an ulcer of 2 x 2 cm with a nodular margin located on the medial wall of the second part of the duodenum near the duodenal papilla (Figure 2). 

**Figure 3:**
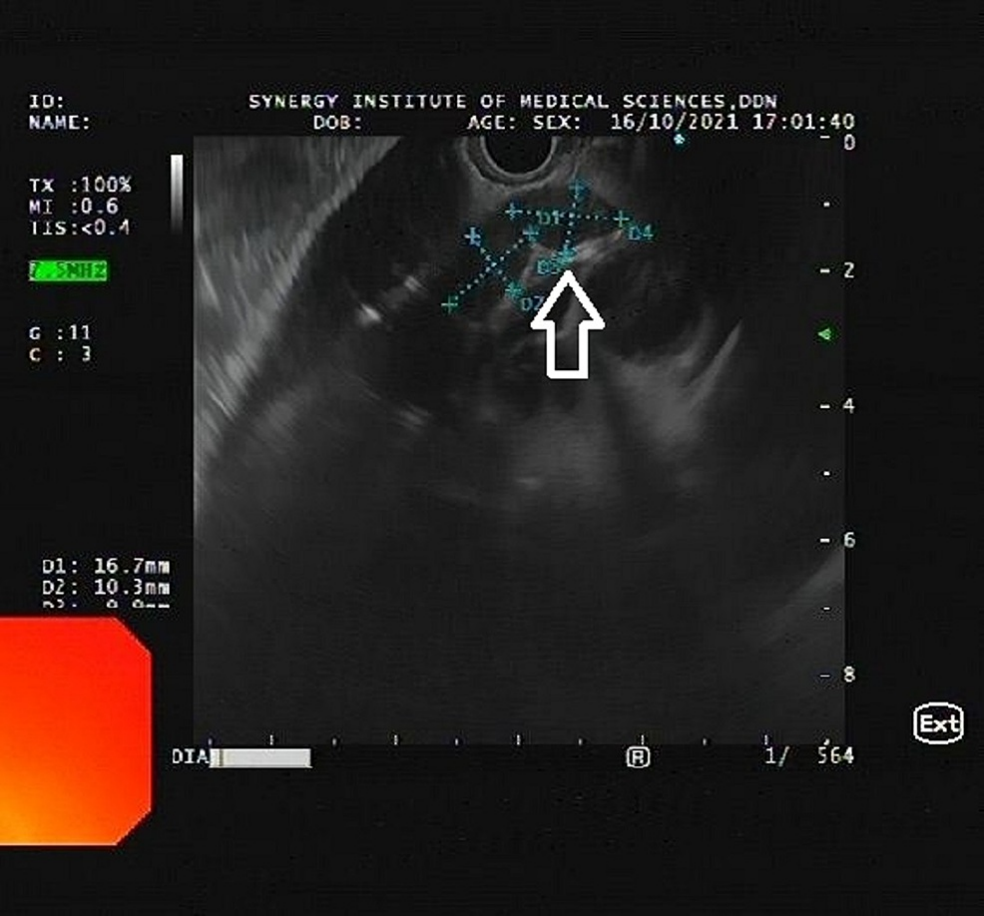
Endoscopic ultrasound showing the enlarged mediastinal lymph nodes (arrow).

Endoscopic ultrasound (EUS) showed multiple heteroechoic lymph nodes in the subcarinal region with the largest diameter of 16 mm (Figure 3).

**Figure 4:**
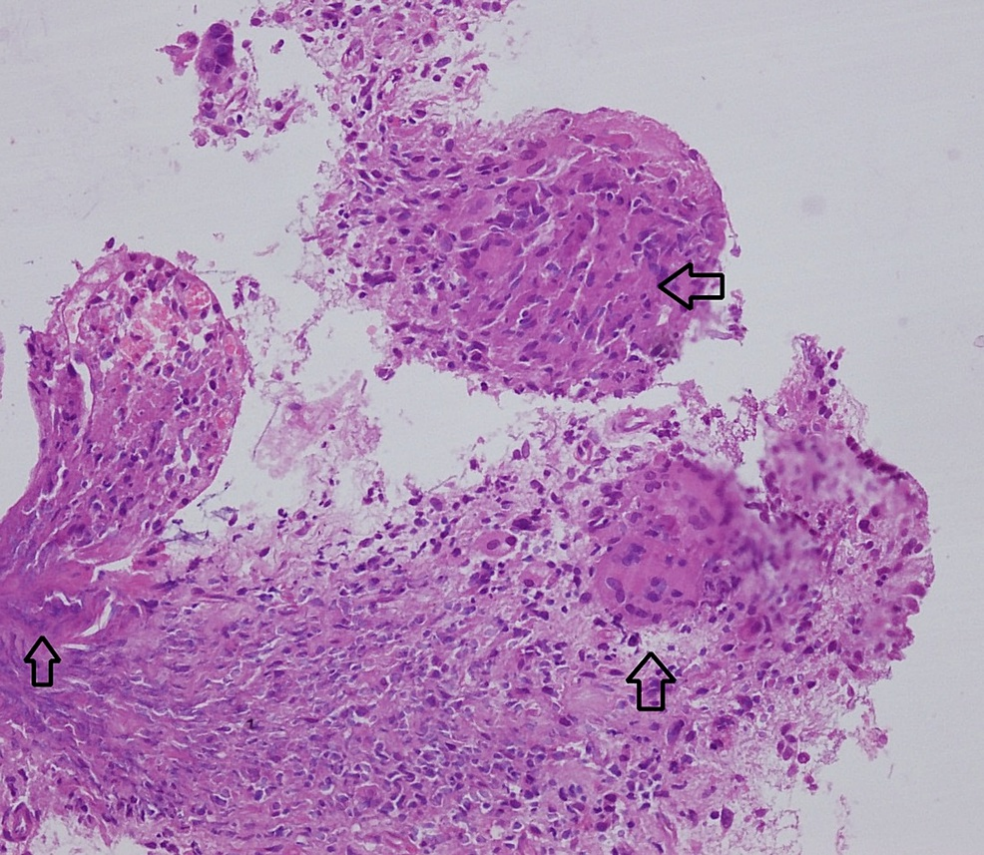
Endoscopic biopsy showed loose aggregates of epitheloid-like histiocytes with presence of Langerhans type of giant cells and granuloma formation (arrows) (H & E, 40x). H&E: hematoxylin and eosin

Additionally, multiple enlarged peripancreatic and portal lymph nodes were also present. The lesion in the pancreatic head could not be clearly visualized on EUS due to the presence of air foci within it. EUS guided fine needle aspiration cytology (FNAC) from the mediastinal lymph nodes showed few degenerated cells with a hemorrhagic background. Initially, the patient received supportive care with correction of electrolyte imbalances. The differential diagnosis based on radiological and endoscopic findings included duodenal malignancy, peptic ulcer with localized retroperitoneal perforation, and groove pancreatitis. Subsequently, the endoscopic biopsy revealed loose aggregates of epitheloid-like histiocytes with the occasional presence of Langerhans type of giant cells and granuloma formation (Figure 4).

Ziehl-Neelsen (ZN) stain and polymerase chain reaction (PCR) for acid-fast bacilli (AFB) was negative. Based on the above findings, the final diagnosis of pancreatoduodenal TB was made. The patient was started on four-drug anti-tubercular treatment (ATT) with isoniazid, rifampicin, ethambutol, and pyrazinamide. On the last follow-up at three months after starting ATT, the patient was symptom-free with weight gain of 5 kg. Repeat endoscopy and CT abdomen will be performed in the subsequent visits to assess the response to ATT.

## Discussion

Gastrointestinal TB is the sixth most frequent site of extrapulmonary TB [10]. Duodenal and pancreatic TB have been reported in about 2.5-4.0% of cases of gastrointestinal TB [10]. Highly acidic environment, rapid transit time, and paucity of lymphoid follicles in the duodenum are the possible reasons for infrequent involvement of the gastroduodenal region in abdominal TB [5,8,11]. 

Duodenal involvement may be external, internal, or both [9,12,13]. The extrinsic type of duodenal involvement is due to enlarged peri-duodenal lymph nodes, while the intrinsic type is due to ulcerative, hypertrophic, or ulcerohypertrophic lesions in the duodenal mucosa. Pulmonary TB may be present in up to 30% of cases of duodenal TB [9]. Duodenal TB may be associated with the involvement of other parts of the gastrointestinal tract in up to 50% of cases [9]. On the other hand, pancreatic TB can present as an abscess, mass, acute or chronic pancreatitis [2,3]. The head region is the most common site of pancreatic TB [14]. 

Males are more commonly affected than females [7,9,12]. Most patients present in the third or fourth decade of life [7,9,14]. Clinical manifestations of pancreatoduodenal TB are non-specific and can mimic other common gastrointestinal diseases such as peptic ulcer, malignancy, and inflammatory bowel disease. Abdominal pain and vomiting are common symptoms of duodenal TB and are reported in about 56.5% and 60.8% cases, respectively [12]. Pancreatoduodenal TB may be associated with fever, weight loss, and palpable epigastric mass as seen in the present case [9,12]. They may present with complications such as ulcer bleed [12], ulcer perforation [4], gastric outlet obstruction, internal gastrointestinal fistula, and obstructive jaundice [6,9,12].

Chest x-ray should be performed in patients with suspected gastrointestinal TB as it may be abnormal in about 20-30% of cases [7]. Abdominal ultrasounds can show hypoechoic pancreatic mass, bowel wall thickening, mesenteric lymphadenopathy, and ascites [7,9]. There are no specific radiologic features of duodenal TB. Traditionally, barium meal follow-through has been the most commonly used radiological imaging for the diagnosis of gastrointestinal TB, especially those involving jejunum, ileum, and colon [12,15]. The predominant findings on barium meal in patients with duodenal TB include duodenal strictures, polypoidal mass, ulcers, perforation, and fistula [9,12,13,15]. Widening of the C-loop of the duodenum or extrinsic compression on the duodenal wall due to pancreatic head TB can also be seen in barium studies in some cases [9]. In abdominal CT, thickening of the duodenal wall, pancreatic mass or abscess, and peripancreatic lymphadenopathy are the most common findings [7,9]. Other CT findings include the presence of necrotic mesenteric or retroperitoneal lymph nodes [6,9,16], ascites [9], jejunal or ileocecal thickening, and dilatation of the intrahepatic biliary ducts [12]. 

UGIE and EUS are the modalities of choice to obtain tissue samples for making the definitive diagnosis of duodenal and pancreatic TB, respectively. Endoscopic findings in duodenal TB include mucosal erythema, nodularity, mucosal thickening, ulceration, stricture, and fistulous opening [12]. On the other hand, the EUS findings in pancreatic TB are the presence of hypoechoic lesions with or without calcifications, common bile duct and main pancreatic duct dilatation, peripancreatic and mediastinal lymphadenopathy, and ascites [16]. However, none of these findings are specific to TB and often present in pancreatoduodenal malignancies. Hence, endoscopic or EUS-guided biopsy should be performed whenever possible to make the diagnosis. The biopsy should be sent for histological analysis, AFB staining/culture, and PCR testing. In a recent systematic review of 166 cases of pancreatic TB, the diagnostic yield of histology, culture, AFB staining, and PCR were 59.7%, 28.9%, 27.7%, and 9.6%, respectively [14].

The treatment of pancreatoduodenal TB is similar to that recommended for extrapulmonary TB. Previous studies have reported high cure rates with standard ATT given for 6-12 months [12,14,16]. Surgical or endoscopic treatment may be required in patients with complications such as gastric outlet obstruction, obstructive jaundice, and gastrointestinal bleeding. However, many reported cases have undergone laparotomy due to the preoperative suspicion of malignancy [12,14]. Hence, preoperative tissue biopsy can prevent unnecessary surgeries as seen in the present case.

## Conclusions

TB is a worldwide problem and diagnosis of TB in rare sites such as the duodenum and pancreas remains a challenge. Pancreatoduodenal TB often mimics other common diseases such as peptic ulcer disease and malignancy. A high index of clinical suspicion is required to make the diagnosis. Radiological, laboratory, and endoscopic investigations can help in making a timely diagnosis. Once diagnosed, ATT should be started to prevent serious complications such as gastrointestinal bleeding and avoid unnecessary surgery. 
